# Fatiguing Exercise Intensity Influences the Relationship between Parameters Reflecting Neuromuscular Function and Postural Control Variables

**DOI:** 10.1371/journal.pone.0072482

**Published:** 2013-08-26

**Authors:** Sébastien Boyas, Anthony Remaud, Erin Rivers, Martin Bilodeau

**Affiliations:** 1 Bruyère Research Institute, Aging and Movement Research Laboratory, Ottawa, Ontario, Canada; 2 University of Ottawa, Faculty of Health Sciences, School of Human Kinetics, Ottawa, Ontario, Canada; 3 University of Ottawa, Faculty of Health Sciences, School of Rehabilitation Sciences, Ottawa, Ontario, Canada; University of Sydney, Australia

## Abstract

The purpose of this study was to investigate the influence of fatiguing exercise intensity on the nature and extent of fatigue-induced changes in neuromuscular function and postural stability in quiet standing. We also explored the contribution of selected neuromuscular mechanisms involved in force production to postural stability impairment observed following fatigue using an approach based on multivariate regressions. Eighteen young subjects performed 30-s postural trials on one leg with their eyes closed. Postural trials were performed before and after fatiguing exercises of different intensities: 25, 50 and 75% of maximal isometric plantarflexor torque. Fatiguing exercises consisted of sustaining a plantarflexor isometric contraction at the target intensity until task failure. Maximal isometric plantarflexor torque, electromyographic activity of plantarflexor and dorsiflexor muscles, activation level (twitch interpolation technique) and twitch contractile properties of plantarflexors were used to characterize neuromuscular function. The 25% exercise was associated with greater central fatigue whereas the 50 and 75% exercises involved mostly peripheral fatigue. However, all fatiguing exercises induced similar alterations in postural stability, which was unexpected considering previous literature. Stepwise multiple regression analyses showed that fatigue-related changes in selected parameters related to neuromuscular function could explain more than half (0.51≤R^2^≤0.82) of the changes in postural variables for the 25% exercise. On the other hand, regression models were less predictive (0.17≤R^2^≤0.73) for the 50 and 75% exercises. This study suggests that fatiguing exercise intensity does not influence the extent of postural stability impairment, but does influence the type of fatigue induced and the neuromuscular function predictors explaining changes in postural variables.

## Introduction

Postural control is a key ability for day to day living and physical activity performance [1]. Information from the visual, vestibular and somatosensory systems is continuously integrated to allow the body to optimally maintain and adjust balance [2]. During quiet standing, the body’s centre of mass falls anterior to the talocrural joint axis. Consequently, ankle muscles, plantarflexors (PFs) and dorsiflexors (DFs), play an active role in sustaining balance, both during bipedal and unipedal stances [3],[4].

Neuromuscular fatigue is a complex phenomenon involving physiological processes occurring in structures from the motor cortex to muscle contractile proteins [5]. Mechanisms of fatigue are influenced by the characteristics of the task being performed, such as the type of exercise (continuous vs. intermittent), the muscles and joint(s) involved, or exercise intensity [6]. Exercise intensity influences the duration of the fatiguing exercise when performed until failure [7]. Indeed, the higher the exercise intensity, the shorter the duration of the fatiguing task. Exercise intensity has also been reported to modify the nature and extent of the physiological changes associated with neuromuscular fatigue [8]. Low-intensity, long-duration exercises are associated with greater central fatigue (impairment of the central command), whereas high-intensity, short-duration exercises involve greater peripheral fatigue (impairment of the mechanisms from muscle excitation to contraction). This has been illustrated by Smith et al. [9], who have reported using transcranial magnetic stimulation, that two thirds of the decrease in force-generating capacity can be attributed to central (supraspinal) mechanisms during isometric flexion of the forearm at 5% of maximal effort. In contrast, Kent-Braun [10] and Schillings et al. [11] have estimated, via twitch characteristics, that only 20 and 12% of the loss of strength are due to central fatigue respectively after a maximal isometric contraction of ankle DFs and arm flexors. Of specific interest to the present study, the literature suggests that fatigue induced by repeated maximal isometric contractions of PFs [12] or alternated maximal isometric PF contractions and sub-maximal isometric DF contractions performed until torque decreases below 50% of maximum for both muscle groups [13] is associated with both central and peripheral fatigue. However, central factors seem to contribute more to the force production decrement of PFs, whereas peripheral factors are seemingly predominant for DFs [13], [14].

Neuromuscular fatigue has been reported to impair postural stability [15]. Because of their important role in balance, the influence of PF fatigue on postural control has been documented in several studies [16], [17]. Various characteristics of fatiguing exercises may induce different changes in postural stability. Indeed, we have reported that fatiguing PFs and DFs simultaneously was associated with greater impairment in postural stability than when the same muscles were fatigued separately [18]. On the other hand, we did not observe any significant difference in postural stability impairment when comparing isometric vs. isokinetic fatigue of the PFs [16]. Exercise intensity could also influence the extent of postural stability impairment due to fatigue. To our knowledge, only Harkins et al. [19] have compared the effects of fatiguing exercise intensity on postural stability. These authors reported that sway velocity increase was greater after isokinetic contractions of the PFs and DFs sustained until torque decreased to 30% compared with 50% of maximum. They also observed that the fatigue-induced increase in sway velocity lasted longer after the 30% fatiguing exercise. It would be interesting to extend these observations by investigating other contraction modes and a broader range of exercise intensities.

Furthermore, the mechanisms responsible for the fatigue-induced impairment in postural stability and their relative contribution remain to be elucidated. Postural stability impairment observed with fatigue could be due to alterations in the transmission or processing of proprioceptive information, changes in muscle/joint stiffness, and/or altered neuromuscular system functioning (i.e., reduced ability to produce an appropriate amount of force with adequate timing [15]). As an initial step, this study focused on the relationships between changes in the later mechanisms (neuromuscular system impairments leading to potential alterations in force production) and changes in postural stability.

The first aim of the present study was to investigate the influence of fatiguing exercise intensity on the nature and extent of fatigue-induced changes in neuromuscular function and on the extent of postural stability impairments. It was hypothesized that exercises of lower intensity would induce more central fatigue than those of higher intensity. We also hypothesized that the presence of central fatigue following low-intensity exercise would lead to greater alterations in the tested postural variables. In order to provide further information concerning the second hypothesis, we sought to document the contribution of selected neuromuscular mechanisms (voluntary activation, muscle contraction capacity) involved in force production to postural stability impairments using an approach based on multivariate regressions. We hypothesized that changes in postural stability with lower-intensity fatiguing exercises would be associated particularly with mechanisms related to central fatigue (e.g. voluntary activation), whereas for higher-intensity fatiguing exercises, the association would be stronger with peripheral factors (e.g. electrically elicited torque).

## Materials and Methods

### Participants

Eighteen (8 men, 10 women) healthy adults (20.9±3.2 years, 171.4±10.2 cm, 67.1±12.7 kg) volunteered for this study. All subjects were informed of the purpose and protocol of the study and each gave formal written consent. The research protocol was approved by the research ethics boards of the Bruyère Research Institute and the University of Ottawa. None of the participants reported any neurological, vestibular disorder or orthopedic condition, including lower-limb injury in the six months prior to data collection. No participant was taking any medication known to influence balance or neuromuscular function.

### Experimental Design

**Figure 1:**
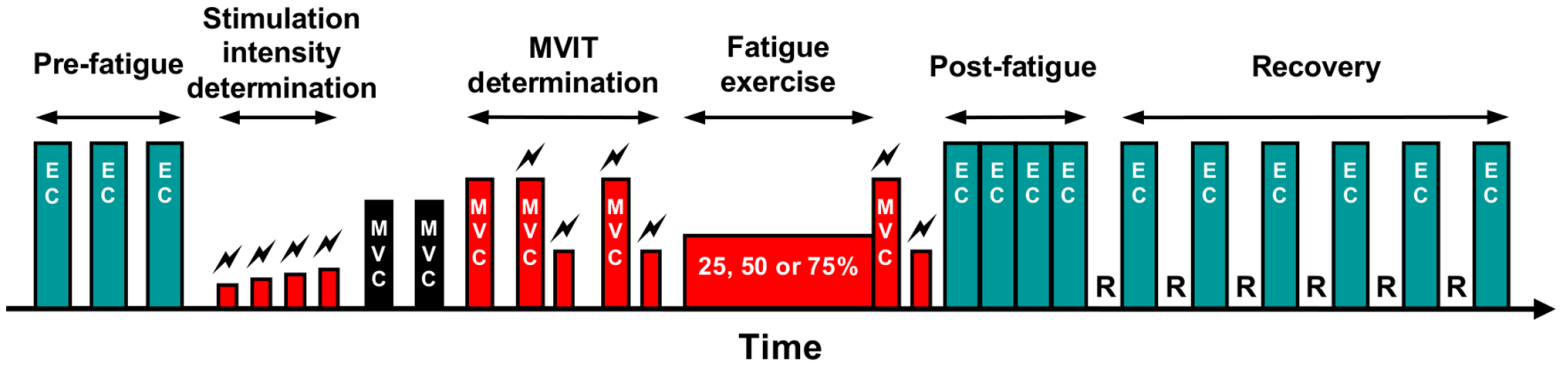
Experimental design. EC: postural trial performed with eyes closed. 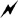: electrical stimulation. MVC: maximal voluntary contraction of DFs (black bars) and PFs (red bars). MVIT: maximal voluntary isometric torque. R: 30-second rest period.

An overview of the protocol for a typical experimental session is given in figure 1. Each subject participated in three experimental sessions, which differed only in the intensity of the fatiguing exercise, i.e. 25, 50, or 75% of the maximal voluntary PFs isometric torque (MVIT). Sessions 1 and 2 were performed on average 4.5±2.8 days apart, and sessions 2 and 3 4.2±1.9 days apart. The order of the experimental sessions was counterbalanced across subjects. Each experimental session was composed of seven steps: setup for electromyographic (EMG) recordings, pre-fatigue postural trials, determination of supramaximal electrical stimulation intensity, determination of MVIT, fatiguing exercise, post-fatigue MVIT, and post-fatigue and recovery postural trials.

#### EMG recording setup

Prior to all testing, EMG surface electrodes were placed on the subject’s right leg in order to record signals from the main superficial muscles involved in ankle plantar flexion, i.e. gastrocnemius lateralis and medialis, soleus, and the main muscle involved in dorsiflexion, i.e. tibialis anterior. Electrodes were placed on the muscles according to SENIAM recommendations [20], with the interelectrode axis aligned with the assumed direction of muscle fibres. Measurements of the exact location of each electrode pair were taken using photographs and a measuring tape to ensure consistent positioning across sessions. Electrodes were applied to the muscles during submaximal contractions to limit the influence of skin displacement on electrode positioning. Electrodes were 1 mm in width, 10 mm in length with a 10 mm center-to-center interelectrode distance (DE-2.1, Delsys Inc., Boston, USA). The common reference electrode was placed on the patella of the right leg. EMG signals were recorded during the entire duration of the experimental sessions using the Bagnoli 16 EMG system (Delsys Inc., Boston, USA) at a sampling rate of 5,000 Hz.

#### Pre-fatigue postural trials

During postural trials, subjects had to stand barefoot on their right leg with eyes closed. We selected a unipedal task because our fatigue protocol only involved single-leg musculature. This task was performed without vision as it has been shown to greatly challenge postural control in young adults (9, 29), thus making this postural task more sensitive to a fatigue effect (7). All postural trials lasted 30 s. Subjects were asked to lift their left leg and establish balance before closing their eyes. Subjects were instructed to remain as steady as possible (i.e., minimize sway) during trials. Subjects had to place their arms at their sides at the beginning of the postural trials but were allowed to use them for balance if necessary. Each subject’s foot placement was clearly marked on the platform to ensure consistency throughout testing. A force platform (Accugait, AMTI, Watertown, USA; sampling frequency of 100 Hz) was used to document center of pressure (COP) displacements before and after fatigue. After familiarization with the postural task, subjects completed three pre-fatigue postural trials with eyes closed (PRE).

#### Determination of supramaximal electrical stimulation intensity

Subjects were then asked to lie supine on a dynamometer chair (Biodex System 3 Pro®, Biodex medical systems, Shirley, USA) with their hips flexed at 25°, the right knee fully extended (0°), and the right ankle positioned at 90°. After a warm-up period, stimulation electrodes (5 cm×2 cm, Chattanooga Group, Hixson, USA) were placed over the posterior tibial nerve (i.e. in the popliteal fossa). Single pulses of electrical stimulation (square-wave stimulus of 200 µs duration with a maximal voltage of 400 V; Digitimer DS7AH, Digitimer Ltd., Hertfordshire, United Kingdom) of increasing intensity were delivered until the plantar flexor twitch torque and the amplitude of the M-wave of the soleus reached a maximal value. The stimulation intensity was then increased further by 20%.

#### Determination of MVIT

Subjects performed two maximal isometric ankle dorsal flexions and three maximal isometric ankle plantar flexions to determine the MVIT produced respectively by the DFs and PFs, and to record the associated maximal EMG activity. Subjects performed one extra maximal plantar flexion to avoid the potential bias (i.e. non-maximal contraction) related to fear of electrical stimulation. Consequently, the first maximal voluntary contraction (MVC) was performed without any stimulation whereas the following two MVCs comprised a superimposed doublet of electrical stimulation at 100 Hz (10-ms interstimulus interval) applied when subjects reached their maximal torque. Two to three seconds after the end of the MVC, another doublet (potentiated) was applied. These MVCs lasted 5 s. During all MVCs, subjects were strongly encouraged.

#### Fatiguing exercise

During each experimental session, subjects performed a fatiguing isometric contraction of the PFs at one of the three intensities: 25, 50, or 75% MVIT. Subjects had to maintain the requested torque level until task failure, which was determined as the inability to sustain the target torque for more than 3 consecutive seconds. Visual feedback of the torque produced was provided to the subjects to help them maintain the requested torque. Strong verbal encouragements were provided during the fatiguing exercises.

#### Post-fatigue MVCs

Immediately after the end of the fatiguing exercise, subjects performed a single MVC of the PFs, which also comprised a superimposed doublet, followed by a potentiated doublet 2–3 s later.

#### Post-fatigue and recovery postural trials

Subjects then performed four post-fatigue postural trials (POST1 to POST4) with only 3–5 s in between. Post-fatigue trials were followed by a 30-s rest and six recovery postural trials (REC1 to REC6), with 30-s rest periods (seated on a chair) between trials.

### Data Analysis and Signal Processing

#### MVIT

The highest torque value recorded during the three MVCs of the PFs was considered as the MVIT.

#### Mechanical responses to stimulation

The following parameters were calculated from the potentiated doublets: (1) peak torque: highest value of doublet torque production; (2) contraction time: time required to reach doublet maximal torque, calculated from the start of the mechanical response to the stimulation; (3) half-relaxation time: defined as the time required for torque to decay from 100% to 50% of the peak torque value.

#### Voluntary activation

The ability of subjects to maximally activate the plantarflexor muscle group through volition was assessed through the level of voluntary activation, as calculated using the following equation [21]:

Activation level (%) = (1– interpolated doublet torque/potentiated resting doublet torque)×100.

#### Postural variables

COP data were used to calculate five postural variables which provided an overview of the subject’s postural stability (i.e. subject’s ability to remain as still as possible and to limit COP displacements): the area of the 95% confidence ellipse (sway area, cm^2^), the mean sway velocities in the antero-posterior (AP velocity, cm.s^−1^) and medio-lateral (ML velocity, cm.s^−1^) axes, and the standard deviation of the COP in the antero-posterior (AP SD, cm) and medio-lateral (ML SD, cm) axes. Considering the fact that subjects were instructed to minimize their sway, sway area reflected the general postural performance (larger sway area corresponding to worse performance), with AP SD and ML SD giving more detailed information for each axis. AP and ML velocities provided information on postural corrections required to maintain postural stability (a higher velocity indirectly reflecting a higher amount of postural corrections).

#### Electromyography

Surface EMG signals were digitally filtered (bandwidth 6–500 Hz) in order to reduce slow transients and instrumentation noise. Then, an estimate of the overall level of activity of the muscle, the root mean square (RMS) value, was calculated over the entire 30-s duration of each postural trial. RMS values were expressed as a percentage of the maximal value recorded during MVCs of the PFs and DFs (% RMS_max_). The RMS_max_ values were calculated on a 0.5 s window around the peak torque, considering the MVC with the highest torque. Care was taken to exclude the electrical response to the doublet in these calculations. The activity of the medial and lateral gastrocnemius was averaged and considered as gastrocnemii activity.

### Statistics

All descriptive statistics presented in the text, tables and figures are mean values ± standard deviation. Statistical analyses were performed using Statistica software (StatSoft Inc., Tulsa, USA).

One-way analyses of variance (ANOVAs) for repeated measures were used to assess the effect of the intensity of the fatiguing exercise (25, 50, 75% MVIT) on the duration of the fatiguing exercise and on the percentage of changes in MVIT, peak torque, contraction time, half-relaxation time and voluntary activation observed after fatigue.

Two-way ANOVAs for repeated measures were used to assess the effects of the intensity of the fatiguing exercise (25, 50, 75% MVIT) and time (PRE, POST) on MVIT, peak torque, contraction time, half-relaxation time, voluntary activation, and muscle activities (gastrocnemii, soleus, tibialis anterior).

Two-way ANOVAs for repeated measures were also used to assess the effects of the intensity of the fatiguing exercise (25, 50, 75% MVIT) and time (PRE, POST1, POST2, POST3, POST4, REC1, REC2, REC3, REC4, REC5, REC6) on postural variables (Sway area, AP and ML velocities and AP and ML SD).

When relevant, *post-hoc* tests were performed by means of Newman–Keuls procedures. When the sphericity assumption in repeated measures ANOVAs was violated (Mauchly’s test), a Geisser/Greenhouse correction was used.

Forward stepwise multiple linear regression analyses were performed in order to explain the fatigue-induced changes in postural variables (sway area, ML and AP velocities and ML and AP SD) from the fatigue-induced changes in MVIT, peak torque, contraction time, half-relaxation time, voluntary activation and muscle activity (gastrocnemii, soleus, tibialis anterior). Selection of the predictors for regression analysis was based on the improvement of the R^2^ value. Predictors were selected in the regression model when their contribution improved the R^2^ value by at least 0.05. For all statistical tests, the significance level was set at 0.05.

## Results

### Characterization of Fatigue

#### Duration of fatiguing exercises

A significant main effect of intensity (F(2,34) = 47.1, p<0.001, η^2^_p_ = 0.73) was found for the duration of the fatiguing exercise. The fatiguing exercise performed at 25% MVIT (643.3±350.4 s) lasted significantly (p<0.001) longer than those performed at 50% (171.7±61.4 s) and 75% MVIT (65.7±27.0 s). There was no significant difference between 50% and 75%.

#### Maximal Voluntary Isometric Torque (MVIT)

A significant time×intensity interaction (F(2,34) = 15.7, p<0.001, η^2^_p_ = 0.48) was found for MVIT. Pre-fatigue MVITs were not different (p>0.05) for the three experimental sessions (table 1). Fatigue induced a significant (p<0.001) drop of the MVIT values after the three experimental sessions. This drop was greater (p<0.001) after the 25% and 50% exercises than after the 75% exercise. The decrease in MVIT was also greater (p<0.05) after the 25% exercise than after the 50% exercise.

**Table 1:** Values of neuromuscular function parameters before and after fatigue according to the intensity of the fatiguing exercise.

Parameter	Intensity	Pre-fatigue	Post-fatigue	% change
**MVIT (N⋅m)**	25	113.3±21.8	79.0±23.7***	−30.2†
	50	109.3±23.1	83.1±18.4***	−24.0‡
	75	110.8±20.7	97.8±18.6***	−11.7
**Doublet peak torque (N⋅m)**	25	34.8±8.6	31.6±7.6***	−9.4
	50	33.3±9.0	31.5±7.8***	−5.6
	75	34.7±8.5	34.2±9.0	−1.5†
**Doublet contraction time (s)**	25	0.125±0.008	0.122±0.012	−1.9†
	50	0.126±0.012	0.153±0.029***	+21.5
	75	0.125±0.008	0.151±0.023***	+20.6
**Doublet half-relaxation time (s)**	25	0.089±0.012	0.120±0.027***	+35.6†
	50	0.088±0.013	0.189±0.040***	+114.9‡
	75	0.095±0.033	0.162±0.053***	+70.1
**Voluntary activation (%)**	25	94.4±9.0	84.0±14.3***	−11.1†
	50	95.3±7.5	92.1±9.9	−3.4
	75	93.9±8.6	93.9±8.2	+0.1

Values are means ± SD. MVIT: maximal voluntary isometric torque.

*** significantly different from pre-fatigue values (p<0.001).

† % change significantly different from the two other intensities (p<0.05).

‡ % change significantly different from 75% (p<0.05).

#### Peak torque

A significant time×intensity interaction (F(2,34) = 3.4, p<0.05, η^2^_p_ = 0.17) was found for peak torque. Pre-fatigue peak torque values were not different (p>0.05) among experimental sessions (table 1). Fatigue induced a significant (p<0.001) drop of the peak torque values after the 25% and 50% exercises only, without any significant difference between these two intensities.

### Contraction Time

A significant time×intensity interaction (F(2,34) = 15.1, p<0.001, η^2^_p_ = 0.47) was found for contraction time. Pre-fatigue contraction time values were not different (p>0.05) among experimental sessions (table 1). Fatigue induced a significant (p<0.001) increase of the contraction time values after the 50% and 75% exercises only, without any significant difference between these two intensities.

#### Half-Relaxation time

A significant time×intensity interaction (F(2,34) = 25.7, p<0.001, η^2^_p_ = 0.60) was found for half-relaxation time. Pre-fatigue half-relaxation time values were not different (p>0.05) among experimental sessions (table 1). Fatigue induced a significant (p<0.001) increase of the half-relaxation time values after the three experimental sessions. This increase was greater (p<0.001) after the 50% exercise than after the 25% and 75% exercises. The increase in half-relaxation time was also greater (p<0.001) after the 75% exercise than after the 25% exercise.

#### Voluntary activation

A significant time×intensity interaction (F(2,34) = 11.3, p<0.001, η^2^_p_ = 0.40) was found for voluntary activation. Pre-fatigue voluntary activation values were not different (p>0.05) among experimental sessions (table 1). Fatigue induced a significant (p<0.001) drop of the voluntary activation values only after the 25% exercise.

### Postural Variables

**Figure 2:**
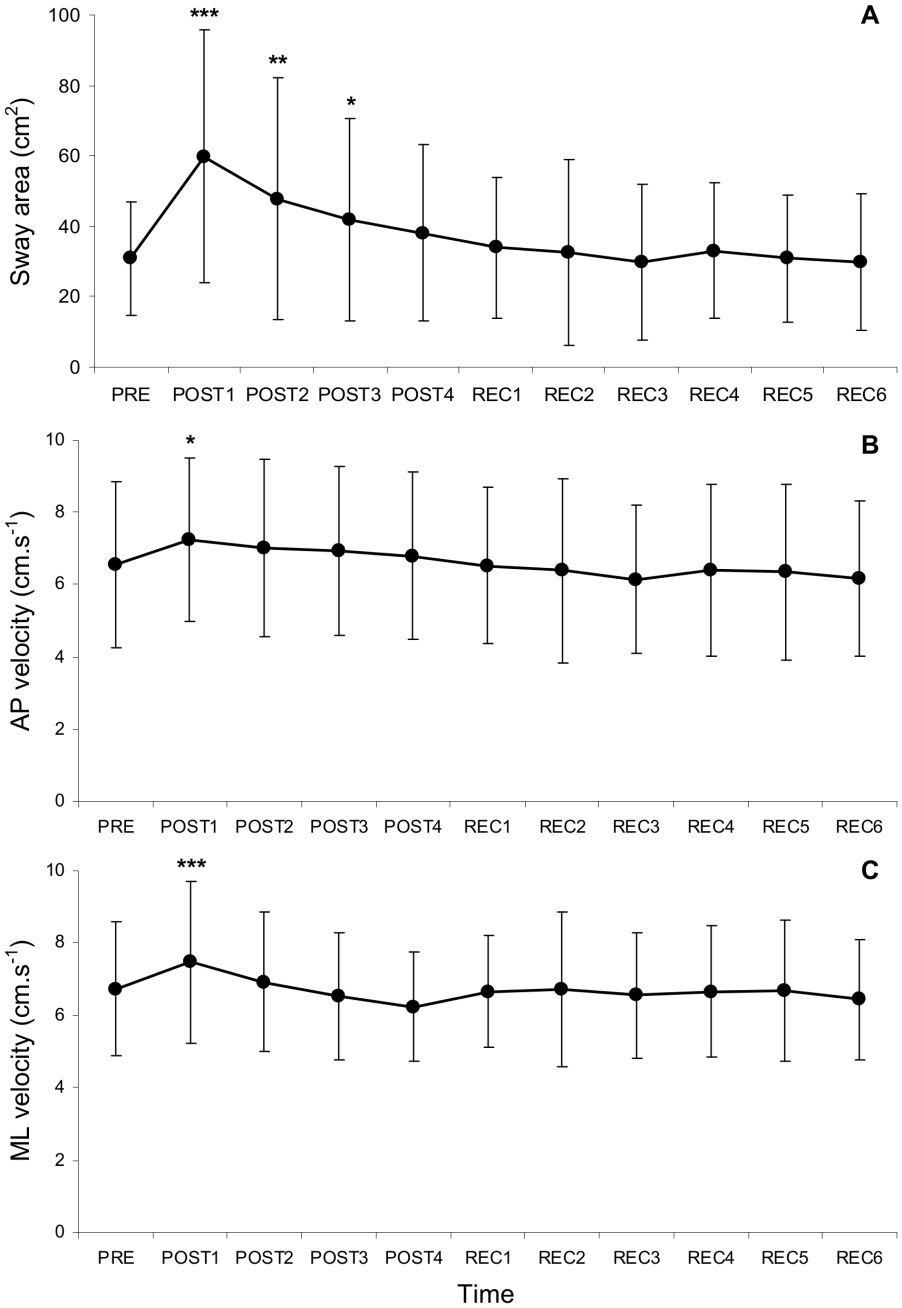
Changes in postural variables over time. A: changes in sway area; B: changes in AP velocity; C: changes in ML velocity. Significantly different from pre-fatigue values: ***: p<0.001, **: p<0.01, *: p<0.05. Values are means ± SD.

No main effect of exercise intensity or time×intensity interaction was found for any postural variables. Therefore, the data presented in figure 2 are pooled from the three exercise intensities.

#### Sway area

A significant main effect of time (F(10,170) = 12.7, p<0.001, η^2^_p_ = 0.43) was found for sway area. Overall, sway area values were respectively 93%, 54%, and 35% greater during POST1 (59.8±35.8 cm^2^; p<0.001), POST2 (47.8±34.2 cm^2^; p<0.01) and POST3 (41.8±28.7 cm^2^; p<0.05) than before fatigue (PRE: 30.9±16.1 cm^2^) (figure 2A).

#### AP velocity

A significant main effect of time (F(10,170) = 4.9, p<0.001, η^2^_p_ = 0.22) was found for AP velocity. Overall, AP velocity values were 10% greater during POST1 (7.24±2.26 cm.s^−1^; p<0.05) than before fatigue (PRE: 6.56±2.29 cm.s^−1^) (figure 2B).

#### ML velocity

A significant main effect of time (F(10,170) = 4.9, p<0.001, η^2^_p_ = 0.22) was found for ML velocity. Overall, ML velocity values were 11% greater during POST1 (7.46±2.23 cm.s^−1^; p<0.001) than before fatigue (PRE: 6.73±1.86 cm.s^−1^) (figure 2C).

#### AP and ML standard deviations

Similar results, i.e. main effects of fatigue, were found for AP (F(10,170) = 9.9, p<0.001, η^2^_p_ = 0.37) and ML (F(10,170) = 9.1, p<0.001, η^2^_p_ = 0.34) standard deviations. Overall, AP SD values were 36% greater during POST1 (1.70±0.55 cm; p<0.001) than before fatigue (PRE: 1.25±0.28 cm). ML SD values were respectively 47% and 33% greater during POST1 (1.83±0.78 cm; p<0.001) and POST2 (1.65±0.74 cm; p<0.001) than before fatigue (PRE: 1.25±0.38 cm).

### Muscle Activity

#### Gastrocnemii

**Figure 3:**
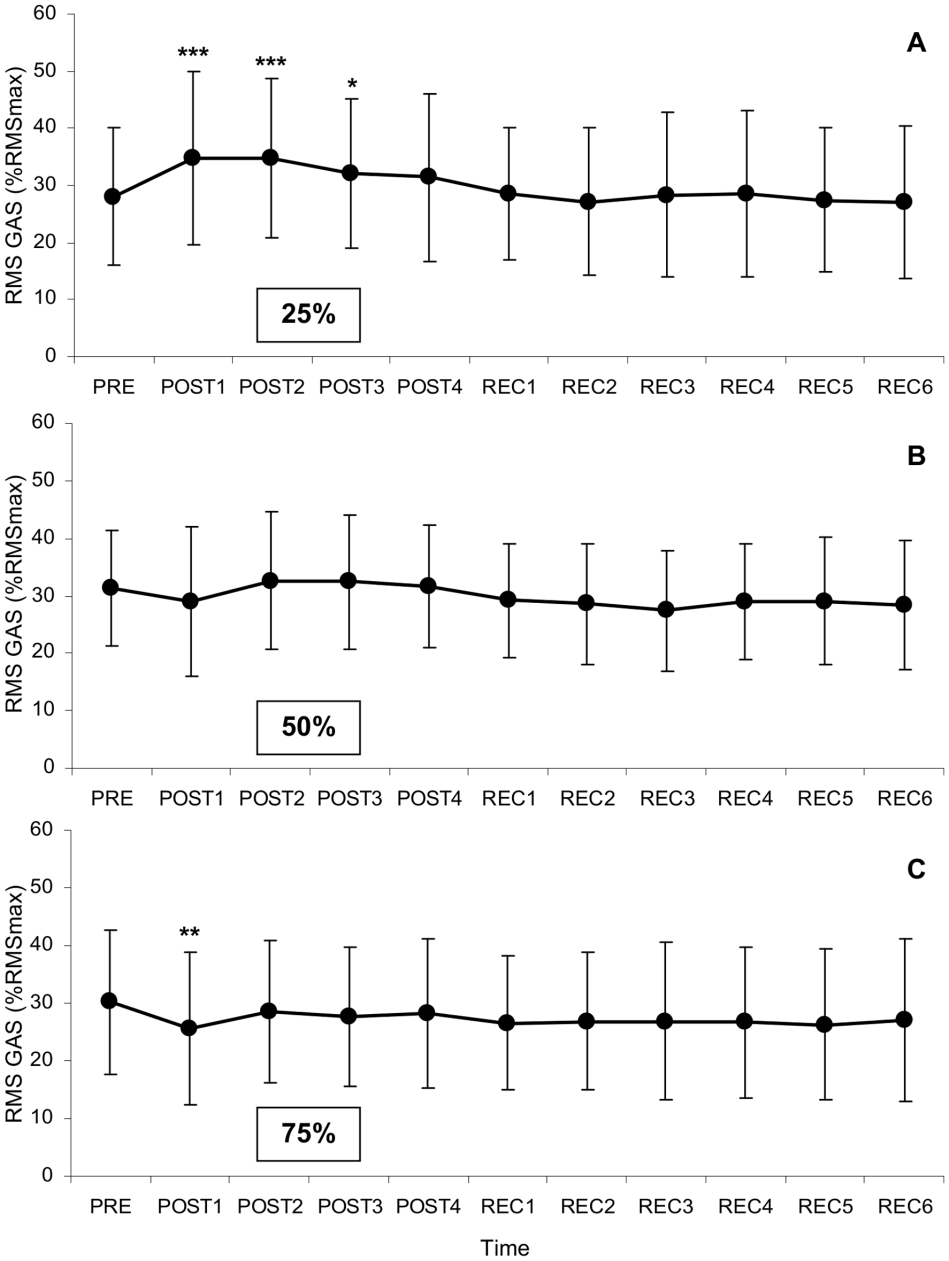
Changes in gastrocnemii muscles activity (RMS) over time for the 25, 50, and 75% MVIT fatiguing exercises. Significantly different from pre-fatigue values: ***: p<0.001; **: p<0.01; *: p<0.05. Values are means ± SD.

A significant time×intensity interaction (F(20,340) = 4.9, p<0.001, η^2^_p_ = 0.22) was found for gastrocnemii muscles activity (RMS). Pre-fatigue RMS values were not different among experimental sessions (figure 3). At 25%, RMS values were greater during POST1 (34.8±15.2%RMS_max_; p<0.001), POST2 (34.9±14.0%RMS_max_; p<0.001) and POST3 (32.0±13.0%RMS_max_; p<0.05) than before fatigue (28.0±12.0%RMS_max_). At 50%, RMS values did not change over time. At 75%, RMS values during POST1 (25.5±13.2%RMS_max_) were statistically lower (p<0.01) than before fatigue (30.2±12.4%RMS_max_).

#### Soleus

A significant main effect of time (F(10,170) = 3.6, p<0.001, η^2^_p_ = 0.17) was found for soleus muscle activity. Overall, RMS values during POST2 (50.6±22.2%RMS_max_) were greater than before fatigue (44.9±17.3%RMS_max_).

#### Tibialis anterior

A significant main effect of time (F(10,170) = 2.7, p<0.01, η^2^_p_ = 0.14) was found for tibialis anterior muscle activity. Overall, RMS values were lower during POST2 (30.3±13.7%RMS_max_) and during REC3 to REC6 than before fatigue (33.7±13.0%RMS_max_).

### Multiple Linear Regression Analyses

Results of the forward stepwise multiple linear regression analyses are presented in table 2. For the 25% MVIT fatiguing exercise, the regression model globally explained more than half of the changes in postural variables (multiple R^2^ ranging from 0.51 to 0.82), whereas it explained less for both other intensities (multiple R^2^ ranging from 0.17 to 0.48 at 50%, and from 0.28 to 0.73 at 75%). For 25%, changes in all postural variables could be explained by a multivariate model that was significant. This was not the case for the other two intensities. It can be noticed that only AP velocity could be explained by significant models for all exercise intensities.

**Table 2:** Multiple linear regressions results explaining the fatigue-induced changes in postural variables from the fatigue-induced changes in neuromuscular parameters (predictors).

Postural variable	Fatiguing exercise intensity							
	25% MVIT		50% MVIT			75% MVIT		
**Δ Sway area**	**Adjusted R^2^**	**Multiple R^2^**		**Adjusted R^2^**	**Multiple R^2^**		**Adjusted R^2^**	**Multiple R^2^**
	0.77	0.81***		0.10	0.21		0.46	0.52**
	**Predictors**	**% R^2^ change**		**Predictors**	**% R^2^ change**		**Predictors**	**% R^2^ change**
	Δ GAS	0.60		Δ MVIT	0.11		Δ VA	0.44
	Δ PT	0.15		Δ CT	0.09		Δ CT	0.09
	Δ MVIT	0.06						
**Δ ML velocity**	**Adjusted R^2^**	**Multiple R^2^**		**Adjusted R^2^**	**Multiple R^2^**		**Adjusted R^2^**	**Multiple R^2^**
	0.45	0.51**		0.36	0.48*		0.18	0.28
	**Predictors**	**% R^2^ change**		**Predictors**	**% R^2^ change**		**Predictors**	**% R^2^ change**
	Δ GAS	0.44		Δ TA	0.29		Δ TA	0.17
	Δ PT	0.07		Δ HRT	0.05		Δ MVIT	0.11
				Δ CT	0.13			
**Δ AP velocity**	**Adjusted R^2^**	**Multiple R^2^**		**Adjusted R^2^**	**Multiple R^2^**		**Adjusted R^2^**	**Multiple R^2^**
	0.50	0.56**		0.37	0.44*		0.32	0.44*
	**Predictors**	**% R^2^ change**		**Predictors**	**% R^2^ change**		**Predictors**	**% R^2^ change**
	Δ GAS	0.46		Δ TA	0.39		Δ SOL	0.28
	Δ SOL	0.10		Δ GAS	0.05		Δ MVIT	0.08
							Δ TA	0.08
**Δ ML standard** **deviation**	**Adjusted R^2^**	**Multiple R^2^**		**Adjusted R^2^**	**Multiple R^2^**		**Adjusted R^2^**	**Multiple R^2^**
	0.56	0.64**		0.11	0.22		0.40	0.47**
	**Predictors**	**% R^2^ change**		**Predictors**	**% R^2^ change**		**Predictors**	**% R^2^ change**
	Δ PT	0.25		Δ MVIT	0.15		Δ VA	0.34
	Δ GAS	0.27		Δ CT	0.06		Δ CT	0.14
	Δ HRT	0.11						
**Δ AP standard** **deviation**	**Adjusted R^2^**	**Multiple R^2^**		**Adjusted R^2^**	**Multiple R^2^**		**Adjusted R^2^**	**Multiple R^2^**
	0.78	0.82***		0.06	0.17		0.67	0.73***
	**Predictors**	**% R^2^ change**		**Predictors**	**% R^2^ change**		**Predictors**	**% R^2^ change**
	Δ GAS	0.67		Δ MVIT	0.10		Δ VA	0.55
	Δ TA	0.08		Δ CT	0.07		Δ PT	0.10
	Δ MVIT	0.06					Δ TA	0.07

*** p<0.001;

** p<0.01;

* p<0.05.

Δ: fatigue-induced changes; MVIT: maximal voluntary isometric torque; PT: peak torque; CT: contraction time; HRT: half-relaxation time; VA: voluntary activation; GAS: gastrocnemii muscle activity; SOL: soleus muscle activity; TA: tibialis anterior muscle activity.

## Discussion

The aim of the present study was to explore the influence of fatiguing exercise intensity on the nature and extent of changes in neuromuscular function and on the extent of postural stability impairment. We also sought to document the relationships between the changes in postural variables and the changes in selected parameters related to neuromuscular function.

Our main results are that the intensity of the fatiguing exercise influenced the nature and extent of neuromuscular fatigue features. The 25% exercise induced more central fatigue than those of higher intensities, whereas the 50% and 75% protocols induced greater peripheral fatigue. However, the intensity of the fatiguing exercise did not influence the extent of postural stability impairment. Multiple linear regression analyses revealed that more than half of the fatigue-induced changes in postural variables could be explained by selected fatigue-induced changes in neuromuscular characteristics of the PFs after the 25% exercise, but less after the 50 and 75% exercises.

### Characterization of Fatigue

Exercise intensity influenced the type (central vs. peripheral) of neuromuscular fatigue induced by the fatiguing protocol. The 25% protocol induced an 11% decrease in voluntary activation, whereas there was no significant change for both other intensities (table 1). A decrease in voluntary activation is an indicator of the presence of central fatigue and indicates that the fatiguing protocol has impaired the ability of the nervous system to maximally activate a muscle or muscle group [5]. The presence of greater central fatigue at the lower exercise intensity validates our hypothesis and is in accordance with the work of Loscher et al. [22] who reported the presence of central fatigue after a sustained isometric contraction of the PFs at 30% MVIT. On the other hand, increases in contraction time and half-relaxation time, which reflect potential impairments in neuromuscular transmission and excitation-contraction coupling and illustrate the presence of peripheral fatigue, were particularly observed after the 50 and 75% protocols (table 1). This result is also in accordance with the literature [10], [11].

Taken together, these results support our hypothesis that the presence of central fatigue is more prominent for low-intensity exercises, whereas the contribution of peripheral fatigue is greater at higher exercise intensities. Our results also indicate that prolonged isometric contractions of the PFs induces both central and peripheral fatigue as reported after repeated maximal isometric contractions [12] or after alternated isometric contractions of PFs and DFs [13].

### Postural Control

The three fatiguing protocols induced changes in postural stability illustrated by increases in all investigated postural variables, i.e. sway area, AP and ML velocities and AP and ML SD. As subjects were instructed to sway as little as possible, increases in postural sway amplitude can be considered a decrease in performance, thus reflecting an impaired ability to efficiently control posture. This fatigue effect was expected and is in accordance with the literature [18], [19]. Fatigue may have induced alterations in both motor and sensory processes, such as altered ability to produce force, increased joint stiffness and impaired proprioception (for a review, see [15]). These alterations would, in turn, lead to inappropriate or delayed stabilizing muscle activation, leading to a less efficient postural control [23].

Although we tested three different exercise intensities over a wide range (25, 50, 75% MVIT), we did not report any significant influence of exercise intensity on the extent of postural stability impairment, which is contrary to our hypothesis. These results are different from those of Harkins et al. [19], who found that COP velocity increased more after a fatiguing protocol of the ankle musculature sustained until maximal torque decreased below 30% than 50% of the initial MVIT. These different results may be due to differences in experimental protocol. First, the contraction mode was isometric in the present study, whereas it was isokinetic for Harkins et al. [19]. As we recently reported, performing isokinetic or isometric fatiguing contractions does not influence the impairment in postural stability when producing a similar level of force reduction [16]. Therefore, contraction mode is probably not the main explanation for the differences observed between our results and those of Harkins et al. [19]. Secondly, in Harkins et al. [19], both PFs and DFs were fatigued, whereas in our study, only the PFs were targeted by the fatiguing protocol. As we illustrated in a previous study, postural stability is impaired to a greater extent when PFs and DFs are fatigued simultaneously than when fatigued separately [18]. The lack of an effect of exercise intensity may suggest that, after fatigue, subjects used non-fatigued muscles of the hip, ankle and/or foot to compensate for the effects of the fatiguing protocol on PFs. Although the type and level of fatigue of the PFs likely differed according to the fatiguing protocol performed (25, 50, or 75% MVIT), this did not lead to significant differences in final postural stability, as reflected by the similar changes in the tested postural variables. Nevertheless, alterations in central and peripheral factors may have resulted in changes (e.g. in the proprioceptive system or in other muscles) that were not measured in the present study. Such changes, which could have differed between the three fatiguing protocols, may have resulted in different sensory reweighting processes or muscle synergies, all of which leading to a similar level of impairment in postural performance (see also next section).

### Associations between Neuromuscular and Postural Stability Parameters

In an initial attempt to provide more direct information on the relative contribution of potential mechanisms responsible for the fatigue-related impairment in postural stability, we used forward stepwise multiple linear regression analyses to document the association between changes in variables reflecting postural stability impairment and changes in selected variables reflecting alterations in neuromuscular function related mainly to force production features of ankle muscles.

First, our results indicated that the use of predictors related to the neuromuscular characteristics of the PFs allowed explaining a significant portion, up to more than 50% of the variance in some cases, of the fatigue-related changes in postural variables, which we think is quite considerable.

Second, it can be observed that predictors reflecting muscle activity (gastrocnemii, tibialis anterior) and voluntary activation were the ones that contributed the most to the significant regression models explaining the changes in postural variables. On the other hand, predictors related to the mechanical response following the electrical stimulation (peak torque, contraction time, half-relaxation time) contributed in a limited way to these models.

Third, exercise intensity influenced our ability to explain the changes in postural variables. Indeed, changes in postural stability were better predicted (higher R^2^) after the 25% fatigue protocol than after the other two protocols of higher intensities (50 and 75% MVIT). The higher R^2^ values observed for the 25% fatigue protocol could be explained by the fact that this protocol induced greater changes in neuromuscular parameters, and in particular, an increase in gastrocnemii muscle activity, which was the predictor that contributed the most to the regression model. We observed that gastrocnemii electrical activity only increased after the 25% protocol. This is probably due to the greater fatigue level of the gastrocnemii after this protocol compared with the other two protocols. This increase in gastrocnemii activity is likely attributable to the recruitment of additional motor units in order to compensate the impairment in the muscle’s capacity to produce the amount of force needed for controlling posture [24], [25]. This increase observed during post-fatigue postural trials could also be explained by bursts of activity (transient recruitment of motor units) of similar amplitude but more frequent. These more frequent bursts of muscle activity would lead to more frequent corrections of the COP position, which would be consistent with the observed increase in mean COP velocity after fatigue. Another explanation for the increase in muscle activity observed in the PFs could be related to an increase in ankle joint stiffness through agonist-antagonist cocontraction [26]. However, the decrease in the activity of the antagonist muscle, the tibialis anterior, does not support this hypothesis.

Concerning the 50 and 75% exercises, the regression models were not as strong as for the 25% exercise, and were less consistent in terms of contributing predictors. For these two intensities, there was no recurrent predictor. Moreover, contributing predictors were a mix of central and peripheral factors. Although traditional COP parameters have been shown to be sensitive to the effect of fatigue intensity on postural performance (Harkins et al., 2005), it is possible that other variables derived from spectral or stochastic analyses, which are more related to the dynamics of postural control, may have led to different results for these two intensities. Also, changes in postural performance at these higher intensities may not be particularly affected by changes in the neuromuscular (motor) system, but more likely by changes in the proprioceptive system [27], [28]. Changes in the neuromuscular system would affect the ability of the muscle to produce the appropriate amount of force for muscle contraction, whereas changes in the proprioceptive system would be related to kinaesthesia, e.g. the ability of the system to detect and integrate changes in joint angles, muscle length, foot pressure. Fatiguing exercises at medium to high intensities would lead to more tension in the muscle-tendon complex and may have led to greater micro-damage to muscles and tendons of the gastrocnemii and consequent alterations in the functioning of the proprioceptors located in these structures that inform on muscle length, tension and joint angle [29]. Such an impairment in proprioceptive function may have led subjects to modify their postural control to maintain balance by using different muscles (such as foot muscles [30]) or by shifting toward a hip strategy [31]. By favoring muscles that are not or less fatigued, more relevant proprioceptive information would be available from these muscles which would result in a more optimized force production and stability. Such a change in postural control would be consistent with the observed decrease in gastrocnemii activity post-fatigue for the 50% and 75% fatiguing exercise protocols.

## Conclusion

In conclusion, the intensity of the fatiguing exercise influenced the type of alterations observed in neuromuscular function. The lower intensity was associated with more central fatigue, whereas higher intensities induced more peripheral fatigue. However, the intensity of the fatiguing exercise did not influence the extent of impairments in postural stability during quiet standing. Variations in predictors identified through regression analyses across fatiguing exercise intensities suggests that different postural control processes were used to achieve the similar level of stability following the different fatigue protocols.
